# Observation of CO Detection Using Aluminum-Doped ZnO Nanorods on Microcantilever

**DOI:** 10.3390/s20072013

**Published:** 2020-04-03

**Authors:** Ratno Nuryadi, Lia Aprilia, Makoto Hosoda, Mohamad Abdul Barique, Arief Udhiarto, Djoko Hartanto, Muhammad Budi Setiawan, Yoichiro Neo, Hidenori Mimura

**Affiliations:** 1 Center for Materials Technology, Agency for the Assessment and Application of Technology, Puspiptek Building #224, South Tangerang, Banten 15314, Indonesia; 2 Research Institute of Electronics, Shizuoka University, 3-5-1 Johoku, Naka-ku, Hamamatsu 432-8011, Shizuoka, Japan; lia7aprilia@gmail.com (L.A.); hosoda.makoto@shizuoka.ac.jp (M.H.); ma.barique@shizuoka.ac.jp (M.A.B.); neo.yoichiro@shizuoka.ac.jp (Y.N.); mimura.hidenori@shizuoka.ac.jp (H.M.); 3 Department of Electrical Engineering, Faculty of Engineering, Universitas Indonesia, Depok, West Java 16424, Indonesia; arief.udhiarto@ui.ac.id (A.U.); djoko@eng.ui.ac.id (D.H.); 4 Center for Technology and Safety of Nuclear Reactor, National Nuclear Energy Agency, Puspiptek, South Tangerang, Banten 15314, Indonesia; setiawan@batan.go.id

**Keywords:** microcantilever sensor, ZnO nanorods, Al doping, resonant frequency shift, CO detection

## Abstract

An oscillating piezoresistive microcantilever (MC) coated with an aluminum (Al)-doped zinc oxide (ZnO) nanorods was used to detect carbon monoxide (CO) in air at room temperature. Al-doped ZnO nanorods were grown on the MC surface using the hydrothermal method, and a response to CO gas was observed by measuring a resonant frequency shift of vibrated MC. CO gas response showed a significant increase in resonant frequency, where sensitivity in the order of picogram amounts was obtained. An increase in resonant frequency was also observed with increasing gas flow rate, which was simultaneously followed by a decrease in relative humidity, indicating that the molecular interface between ZnO and H_2_O plays a key role in CO absorption. The detection of other gases of carbon compounds such as CO_2_ and CH_4_ was also performed; the sensitivity of CO was found to be higher than those gases. The results demonstrate the reversibility and reproducibility of the proposed technique, opening up future developments of highly sensitive CO-gas detectors with a fast response and room temperature operation.

## 1. Introduction

Microcantilever (MC) sensors are MEMS (microelectrochemical system) devices used to detect chemical, physical, and biological objects with high sensitivity, rapid response, and operation at room temperature [1,2,3]. The working principle of this sensor is based on a deflection of the MC due to an object becoming attached to its surface (static mode), or a resonant frequency change of the vibrated MC due to the added mass of the target molecules (dynamic mode). 

In early development of MC-based chemical sensors, physical and environmental parameters such as relative humidity [4], mercury adsorption [5], alkanethiol self-assembled monolayers (SAMs) [6], gases and vapors [7] were measured. In order to detect a specific target, a sensitive layer such as a polymer [8,9] and metal oxide [10,11] needs to be coated on the MC surface. So far, carbon monoxide (CO) gas, which is an odorless, colorless, tasteless, and toxic compound, has been successfully detected using the MC coated with sensitive layers of poly(ethylene oxide)/Ni acetate (PEO/NiA) [8], Fe(III) porphyrin [9], and zinc oxide (ZnO) layers [11,12]. Kooser et al. reported CO detection by PEO/NiA-coated MC, where for small CO exposures, the sensitive layer was fully recoverable; larger CO exposures resulted in permanent material degradation [8]. Reddy et al. demonstrated a piezoresistive MC coated with Fe(III) porphyrin for CO sensing [9]. Our previous experiment assessed CO detection using a ZnO-coated MC at room temperature and found that the presence of water vapor plays a role in the detection mechanism [11]. More recently, we reported the effect of aluminum (Al) dopant atoms in ZnO nanorods on CO adsorption, showing that Al doping significantly increased the sensitivity [12]. Al doping in ZnO leads to a decrease in the HOMO-LUMO energy gap [13] and an increase in the gas adsorption as a result of the smaller atomic size of Al^3+^ ion compared to that of Zn^2+^ (r Al^3+^ = 0.054 nm and r Zn^2+^ = 0.074 nm) [14]. Al-doped ZnO nanorod-coated MC successfully reached a sensitivity in the order of picogram amounts or less, but for many CO monitoring applications in the fields of environment (air pollution and greenhouse emissions) and industry (chemical, civil and transportation industries), further studies considering the effect of humidity and various other gases are still necessary. 

In this study, we evaluate the performance of Al-doped ZnO nanorod-coated on a piezoresistive MC and an uncoated MC for the detection of CO under various gas flow rates at room temperature. The morphological structure, gas flow rate effect, response time, humidity effect, and comparison with other gases were investigated.

## 2. Experimental Methods

**Figure 1:**
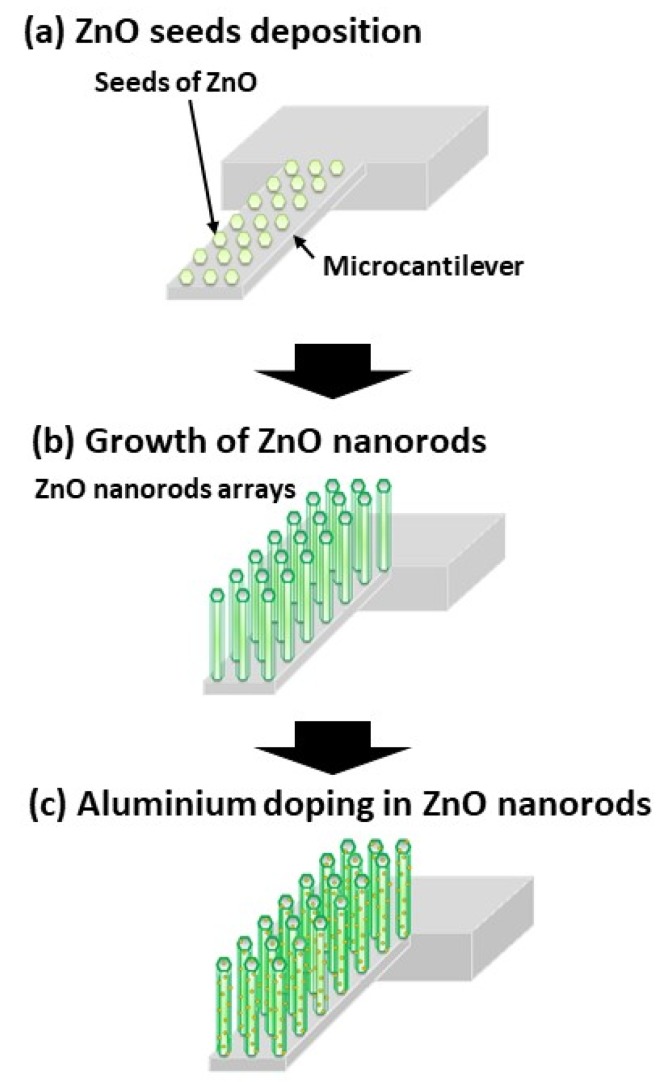
Growth process of Al-doped ZnO nanorods on microcantilever consisting of (**a**) ZnO seeds deposition; (**b**) the growth of ZnO nanorods; (**c**) Al doping in ZnO nanorods using RF sputtering.

Commercially available piezoresistive MCs (NPX1CTP003, Seiko Instrument Inc., Japan) with a spring constant of 4 N/m were used in this study. These MCs consist of two cantilevers with a typical piezoresistor resistance of 630 Ω, i.e., a long cantilever (400 µm long, 50 µm wide, 1 µm thick), which is used to detect the target molecules, and a short one (30 µm long, 50 µm wide, 1 µm thick), which serves as a reference. Note that the reference cantilever is crucial to reduce background noise such as thermal drift and turbulence around the MC. Al-doped ZnO nanorods were grown directly on the MC surface using the hydrothermal method [15,16] through three steps, as shown in Figure 1. First, seeds of ZnO (30-nm thick) were deposited on the MC surface by the radio frequency (RF) sputtering method (Figure 1a). Second, ZnO nanorods were then grown at 60 °C for 2 h by dipping the substrate into a mixed solution of 0.04 M zinc nitrate hexahydrate and hexamethylenetetramine in deionized water (Figure 1b). The ZnO nanorods were grown on the whole surface of the microcantilever during dip process. The grown ZnO nanorods were then dried at 90 °C for 30 min in an oven to evaporate the solvents. Third, Al was doped on the ZnO nanorod-coated MC by RF sputtering with a sputtering time of 105 s (Figure 1c). In Al sputtering, at an RF power of 100 W, argon ions initiate energetic Al atoms from pure Al material. Then, with high kinetic energy induced by the thermal energy from the high temperature heater of the sputtering apparatus (1500 K), the Al atoms bombarded the ZnO nanorod surface to achieve Al doping [12].

**Figure 2:**
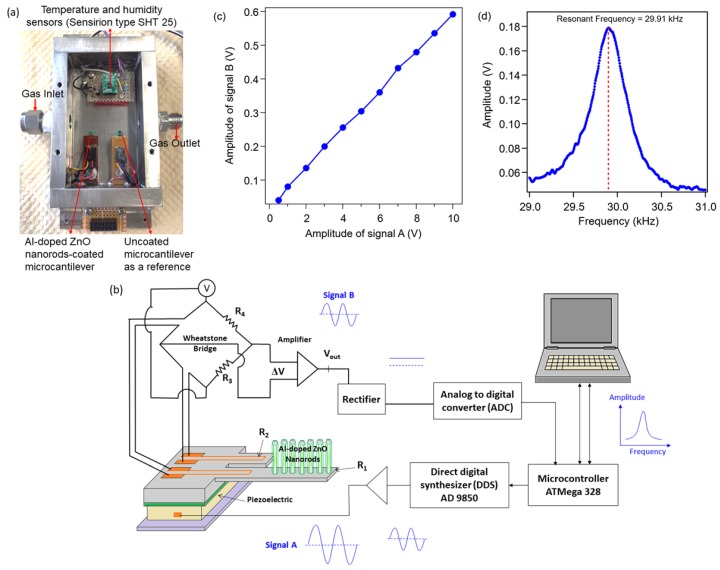
(**a**) View of the inside chamber box made of stainless-steel with a size of 8 cm × 5 cm × 2 cm; (**b**) resonant frequency measurement system based on a microcontroller-controlled direct digital synthesizer (DDS); (**c**) amplitude of signal B as a function of signal A, which vibrates the microcantilever with a resonant frequency of 36.2 kHz; (**d**) profile of the vibration amplitude vs frequency for Al-doped ZnO nanorod-coated MC.

The Al-doped ZnO nanorod-coated MC was then placed on a piezoelectric inside a stainless-steel chamber box with a size of 8 cm × 5 cm × 2 cm, as shown in Figure 2a. An uncoated MC was set next to the Al-doped ZnO nanorod-coated one as a reference sample. The temperature and relative humidity (RH) inside the chamber were monitored by a hygrometer (Sensirion, type SHT 25), which was fully calibrated with 1.8 % RH accuracy. Holes of 9 mm diameter were made on left and right wall sides and connected with pipes to serve as the as gas inlet and outlet. After the preparation of the measurement was finished, the chamber was closed, and hence, gas only flowed through inlet and outlet. CO was introduced into the chamber at a flow rate of 20–100 mL/min. Here, an influence of gas flow on CO response was observed without any other treatment, such as air/nitrogen flashing. 

The MC response, i.e., the change in resistivity of piezoresistor in the long MC, was monitored as output voltage from a Wheatstone bridge circuit consisting of two piezoresistive MCs and two balance resistors, as shown in Figure 2b. Since the MC was operating in dynamic mode, the vibration of the cantilever was activated by applying AC voltage (signal A) to the piezoelectric generated by a microcontroller-controlled direct digital synthesizer (DDS) [12]. This system is fully digital, with the exception of analog circuits in the Wheatstone bridge composed of high S/N-ratio OP-AMPs. The sinusoidal output voltage (signal B) from the Wheatstone bridge contributed to the change in resistivity of the piezoresistor due to the vibration of the MC [17]. The amplitude of signal B was measured using a rectifier circuit and converted with a 16-bit analog to digital converter (ADC) with several hundred averaging operations to smooth out fluctuations in the analog signals. The digital data were then sent to a computer to determine the vibration amplitude vs sweep frequency profile, showing a resonant frequency peak. Figure 2c shows the amplitude of signal B as a function of signal A when the microcantilever with a resonant frequency of 36.2 kHz was vibrated by signal A with the same frequency. As shown, the amplitude of signal B was almost proportional to the amplitude of signal A. Our data were obtained by the measurement setup of the signal B amplitude < 0.5 V; therefore, the experimental results are not influenced by the limitation of the piezoresistor’s resistivity.

The vibration amplitude vs frequency profile of the Al-doped ZnO nanorod-coated MC can be seen in Figure 2d, showing the resonant frequency of 29.91 kHz. Response to the CO gas exposure was detected by measuring the change in the resonant frequency under various gas flow rates. Changes in resonant frequency due to gas response under various initial RHs (environment humidity) on different days were also evaluated. All measurements were performed at room temperature, i.e., 23–25 °C. 

## 3. Results and Discussion

**Figure 3:**
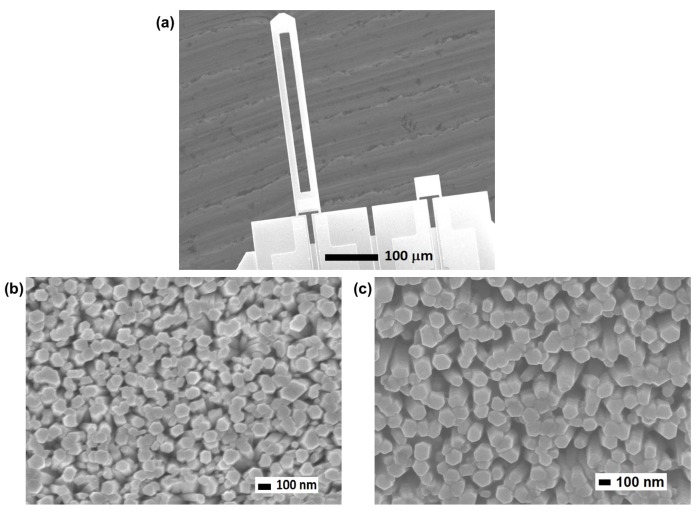
(**a**) Field emission-scanning electron micrograph of the microcantilever and magnified images of ZnO nanorods on the MC surface for (**b**) upper surface and (**c**) lower surface taken by JEOL JSM-7600F, Japan at an accelerating voltage of 5 kV. A vertical nanorod structure and hexagonal shape with diameter of around 100 nm were observed.

Figure 3 shows field emission-scanning electron microscope images of the MC after deposition of the Al-doped ZnO nanorods on the MC surface. The top image was taken with a low magnification; a magnified image of the MC surface is presented below. In Figure 3a, two MCs, i.e., the short cantilever and the long one, are clearly seen. The nanorod structure with an average diameter of 100 nm and an average density of 87 rods/µm^2^ is shown in Figure 3b,c, i.e., Figure 3b for the upper surface of the MC and Figure 3c for the lower surface. The presence of Al-doped ZnO nanorods on both the upper and lower surfaces of the MC indicates that Al-doped ZnO nanorods were grown on whole MC surface in the applied dip process. The hexagonal shape of the nanorods, caused by the behavior of ZnO crystals, can also be seen. From these results, it is clear that the ZnO nanorods were successfully formed on the MC surface. It should be noted that, from the observation of an electron probe micro analyzer (not shown), although Al was unevenly localized on the surface, most of the Al was found to be spread out on the ZnO nanorods. A high concentration of Al occurred where the ZnO nanorods were located, which indicated that Al was selectively bonded with the nanorods. 

**Figure 4:**
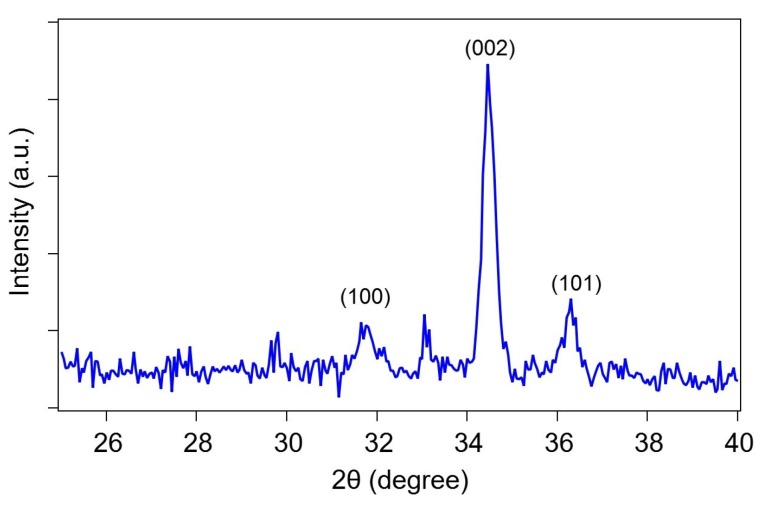
X-ray diffraction pattern of Al-doped ZnO nanorods on the microcantilever surface using a Rigaku Ultima-III X-ray diffractometer with CuK radiation (λ = 0.15418 nm) operated at 40 kV and 40 mA. The strong (002) peak indicates that the ZnO growth direction tends to be oriented along the vertical axis.

Figure 4 shows the X-ray diffraction pattern of the Al-doped ZnO nanorod-coated MC surface taken at room temperature using a scintillation counter operated in a stepping mode with a sample-to-detector distance of 30 cm. It can be seen that by sweeping the diffraction angle 2θ axis from 20° to 40°, three strong peaks were observed at 31.709°, 34.384°, and 36.243°, which attributed to the (100), (002), and (101) ZnO plane [18]. Such peaks, especially the strong (002) peak, also indicate that the ZnO growth direction tends to orient itself along the vertical axis [11,19,20]. As reported in a previous paper [12], the intensity of the (002) peak for Al-doped ZnO nanorods is remarkably smaller than that for pristine ZnO nanorods, indicating that Al doping results in a decrease in crystallinity. The observed lattice constant was estimated to be 3.5024 Å, which is close to the bulk ZnO standard of about 3.253 Å [21]. 

**Figure 5:**
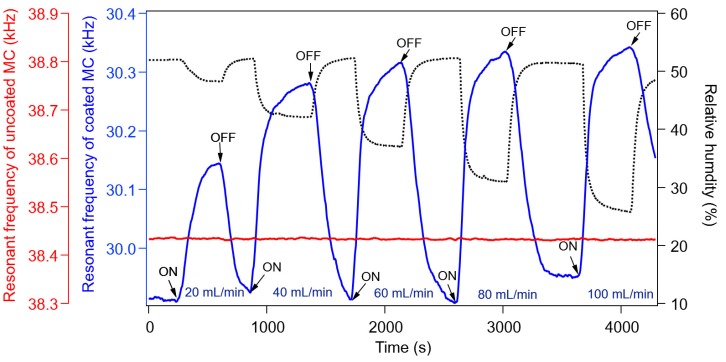
Response characteristics of Al-doped ZnO nanorod-coated MC showing an increase of resonant frequency (blue solid line) and a decrease in RH (black dotted line) due to CO exposure. The increase of resonant frequency and, at same time, the decrease of RH, were also observed with increasing the gas flow rate from 20 mL/min to 100 mL/min. For the uncoated MC (red solid line), there was no response to gas exposure.

We measured the response of the MC to different flow rates of CO. Figure 5 shows a typical plot of Al-doped ZnO nanorod-coated MC resonant frequency (blue solid line) as a function of time for various CO flow rates in the range of 20–100 mL/min. The resonant frequency of the uncoated MC (red solid line) and the relative humidity (RH) (black dotted line) were also measured. An increase in the resonant frequency of the MC was detected when the CO flow was turned on in the chamber. On the other hand, the RH decreased when the gas flow was on. This experiment was performed multiple times, and the results showed reproducibility. It was observed that, generally, the resonant frequency of MC decreases due to the added mass of the target molecules. However, as shown in Figure 5 (blue solid line), the resonant frequency of the Al-doped ZnO nanorod-coated MC increased due to CO exposure. We also confirmed that such a resonant frequency change was not observed at 0 RH (under vacuum condition) [12]; hence, we suggest that the interaction between water vapor and CO molecules plays a role in CO adsorption. The role of water vapor on CO adsorption and the decrease of RH due to gas response is discussed later.

It can also be seen in Figure 5 that the resonant frequency of the uncoated MC (red solid line) was almost constant with the gas flow turned either on or off, indicating that Al-doped ZnO nanorods work properly as a sensitive layer to CO response. Another important piece of data is that an increase in resonant frequency was also observed with increasing the gas flow rate (concentration). It was observed that a saturation of the frequency was reached after 350 s to 400 s, depending on the gas flow rate. Fast saturation was obtained for higher flow rates, which agreed with our previous experimental results [12]. Therefore, such saturation is caused by a saturation of CO concentration. Next, the sensor sensitivity (*dm∕df*) was calculated from the resonant frequency change *df*, as shown in Figure 5. Assuming that *df* ~ 350 Hz for a gas flow rate of 40 mL/min, the mass change *dm* is estimated to be 3 ng, so the sensitivity was determined to be 7.57 pg/Hz.

**Figure 6:**
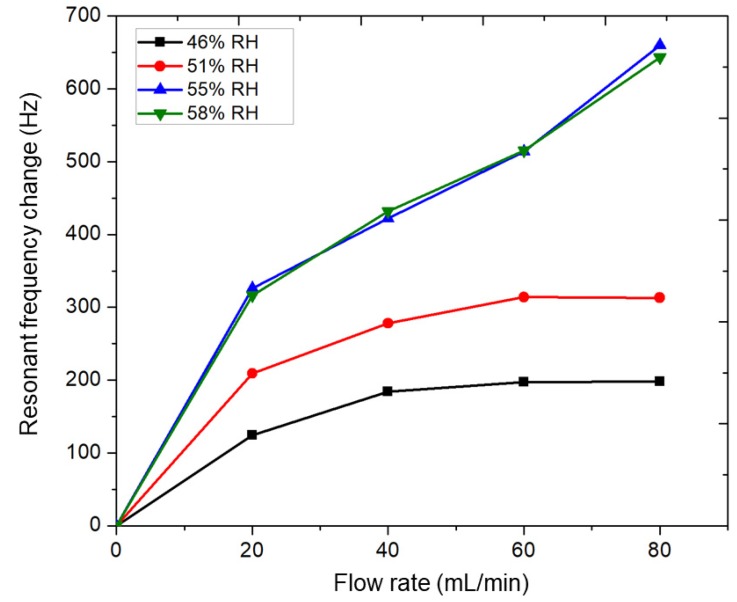
Resonant frequency change of the Al-doped ZnO nanorod-coated microcantilever as a function of gas flow rate for various initial relative humidity (RH) inside the chamber, i.e., 46 %, 51 %, 55 %, and 58 % RH.

The change in the resonant frequency of the Al-doped ZnO nanorod-coated microcantilever as a function of gas flow rate is plotted in Figure 6 for various initial RH inside the chamber before measurement. It was observed that the initial RH inside the chamber before measurement was almost the same with the environment humidity outside the chamber. It was found that the resonant frequency change increased significantly with increasing the CO flow rate. For initial humidities of 46 % and 51 % RH, the change in resonant frequency at 60 and 80 mL/min CO was almost the same; however, the change appeared at 55 % and 58 % RH. Mostly, the resonant frequency change of the MC increased by increasing the water vapor content. The increasing resonant frequency of the MC in Figure 6 is probably due to the lighter total mass of the adsorbed CO molecules compared to the total mass of the water vapor layers on the Al-doped ZnO nanorod-coated MC surface [12]. 

**Figure 7:**
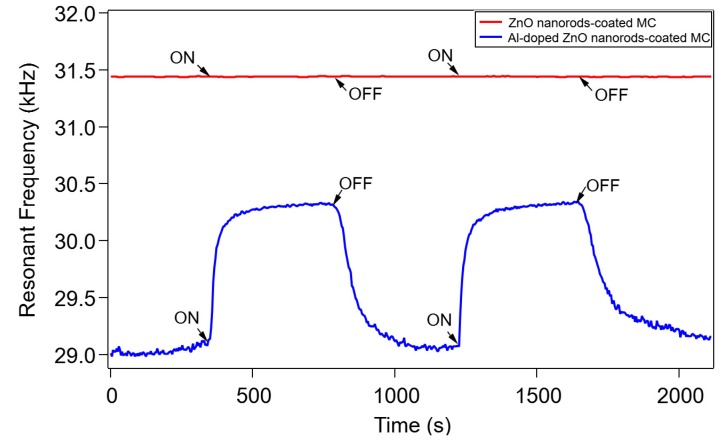
Comparison of CO response between Al-doped ZnO nanorod-coated MC (blue solid line) and undoped ZnO nanorod-coated MC when CO gas was flow either on, with flow rate of 200 mL/min, or off. The graph also shows no resonant frequency change for the undoped ZnO nanorod-coated MC (red solid line) upon gas exposure.

In order to evaluate the effect of Al doping on the CO gas response, we compared the experimental results of the CO gas response for Al-doped ZnO nanorod-coated MC and undoped ZnO nanorod-coated MC, as shown in Figure 7. The experiment was done with CO gas flow on, with a flow rate of 200 mL/min, or off. It can be seen that a change in the resonant frequency (about 1.1 kHz) of the Al-doped ZnO nanorod-coated MC occurred due to gas exposure, while the undoped ZnO nanorod-coated MC showed no gas response. This result indicates that in addition to Al doping in ZnO reducing the HOMO-LUMO energy gap between Al-doped ZnO nanorods and CO, a stronger molecular interaction between CO and the Al-doped ZnO surface occurred, making the π bond easy and leading to an increase in gas adsorption [13].

**Figure 8:**
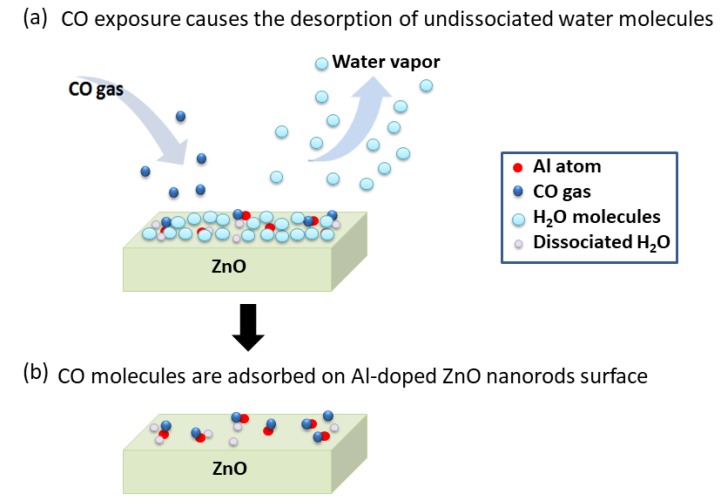
The possible mechanism of CO adsorption on Al-doped ZnO nanorod-coated microcantilever surface. Here, Al atoms (red circles in Figure 8) replaced Zn atoms on the topmost layer of the slab due to the fact that bonding between Zn and O in ZnO crystal lattices is weaker than that those between Zn–Al [13]. (**a**) CO exposure causes the desorption of undissociated H_2_O molecules, and (**b**) simultaneously, CO molecules are absorbed on the Al-doped ZnO nanorod surface.

A possible mechanism of CO adsorption on the Al-doped ZnO nanorod-coated microcantilever surface is proposed in Figure 8. This mechanism is supported by the experimental data, as follows. First, the resonant frequency of the Al-doped ZnO nanorod-coated MC increased due to CO gas exposure (blue solid line in Figure 5), while the resonant frequency of uncoated MC was unchanged (red solid line in Figure 5). This means that Al-doped ZnO nanorods work as a sensitive layer to absorb CO gas. Since the resonant frequency is inversely proportional to the total mass of the MC, this also indicates that the total mass of the MC decreases during CO gas exposure. Second, the change in resonant frequency of the MC only occurs under measurements taken in humid air conditions at room temperature (Figure 5, Figure 6 and Figure 7); the resonant frequency was almost unchanged under 0 RH (vacuum condition) [12]. This indicates that the interaction between the water vapor and CO molecules plays a role in CO adsorption. Third, a higher gas flow rate resulted in a greater increase in resonant frequency (blue solid line in Figure 5) and a larger decrease in RH inside the chamber (black dotted line in Figure 5). This means that the number of CO molecules has a direct correlation with the number of water vapor molecules and the total mass of the MC. Fourth, the change of resonant frequency only occurs for Al-doped ZnO nanorods; almost no CO gas response was observed for the undoped ZnO nanorods sample (Figure 7). This supports the data that Al doping in ZnO nanorods enhances gas adsorption.

In Figure 8a, initially at room temperature, all the Al-doped ZnO nanorods are generally covered by water vapor due to humidity [11,22,23,24,25], where H_2_O (water molecules) are adsorbed either on the Zn [26,27], O [28], or Al-doped site [29]. In such conditions, some water molecules are dissociated into H^+^ and OH^−^ ions [12,22]. Since the surface with the dissociated water is unstable [22], when CO gas enters the chamber, the undissociated H_2_O molecules are desorbed from the surfaces of the Al-doped ZnO nanorods, while CO molecules are absorbed there. The desorption of the undissociated H_2_O molecules and flow of those from the chamber are indicated by the decreasing relative humidity (RH), as shown in Figure 5. Here, most of the CO molecules are adsorbed at Al-doped sites (red circles in Figure 8) due to the lower HOMO-LUMO energy gap between the Al-doped ZnO nanorods and the CO, allowing π bonds to occur more easily and increasing the gas adsorption [13]. It was predicted that the C-heads of the CO molecules would be adsorbed on Al-doped sites due to the shorter distance between the Al atom in the ZnO crystals and the C-head of CO compared to the O–head [13,28]. Almost all the CO molecules are adsorbed on the surface, desorbing the water vapors, as shown in Figure 8(b). A CO molecule is heavier than a H_2_O one, but when the total weight of the adsorbed CO molecules is lighter than that of the desorbed water vapors, the total mass of the microcantilever is reduced, allowing it to vibrate faster, resulting in the increase in the resonant frequency, as shown in Figure 5. 

In our experiment, during CO gas exposure, the air and the CO gas inside the chamber could exit the chamber through outlet pipe and the presence of holes for cable lines. This also allowed the air (containing water vapor) outside the chamber to enter when the gas was switched off. Therefore, the chamber is quasi-opened, and in such conditions, the entering of the CO gas caused the desorption of undissociated water molecules and the RH decreased. Conversely, the increase of RH in Figure 5 occurred due to air entering from outside via the outlet pipe and the cable’s holes when the gas flow was switched off. Moreover, the result in Figure 7 also supports the hypothesis that the resonant frequency change was not caused by a simple H_2_O adsorption–desorption model due to the flow of gas, but rather, by the desorption of H_2_O which was followed by the adsorption of CO molecules on the ZnO surface. In order to confirm this, we also performed Al sputtering on a raw microcantilever and on a ZnO-sputtered microcantilever. We then measured their CO responses, which turned out to be very low, and a large gas concentration was needed for detection. These facts indicate not only the importance of Al bonding in the ZnO nanorod crystal structure, but also the enhancement of CO adsorption due to Al doping after the desorption of H_2_O.

**Figure 9:**
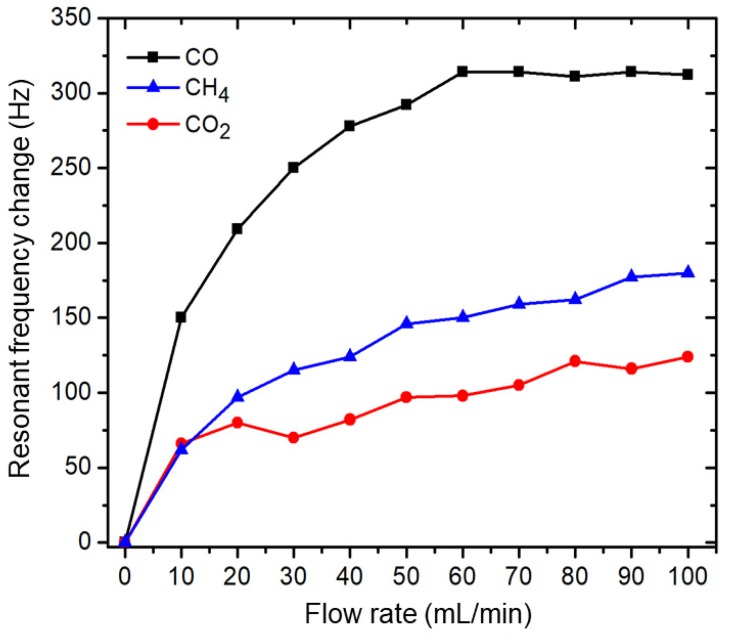
Resonant frequency change of Al-doped ZnO nanorod-coated MC upon exposure to CO, CO_2_, and CH_4_ with flow rates of 10–100 mL/min.

To measure the sensor selectivity, several types gases were measured. Figure 9 shows a comparison of the resonant frequency change for CO, CO_2_, and CH_4_ exposure, measured at flow rates of 10–100 mL/min, which represent varied gas densities/concentrations. CO induced the highest resonant frequency change; the resonant frequency change for CH_4_ was nearly half that of CO, and that of CO_2_ was even lower. CO adsorption-changed resonant frequencies were about 150–300 Hz with a gas flow of 10–60 mL/min; however, saturation was reached with flow rates above 60 mL/min. The results showed the highest sensitivity for CO. The interaction between the HOMO and LUMO for the above gas molecules and Al-doped ZnO nanorods depends on their energy gap (*E_g_*), where a smaller energy gap indicates higher interaction. From the viewpoint of differences in the molecular geometrical configuration of these gas molecules [30], we suggest that the HOMO-LUMO energy gap between CO and the Al-doped ZnO nanorods is comparatively lower than that of CH_4_ and CO_2_, which explains the higher sensitivity for CO as compared to CH_4_ and CO_2_. The adsorption sites for CH_4_ and CO_2_ are probably similar to those for CO, i.e., the Al-doped site, which makes a bond with the C–head of CH_4_ and CO_2_. In addition, the comparison of the responses to the three gases in Figure 9 showing different resonance frequency changes also indicates the occurrence of gas adsorption on the Al-doped ZnO nanorod surface with different levels of sensitivity. 

We calculated the CO concentration or *C_g_* (ppm) using Equation (1) [31]:

(1) $$C_{g}=\frac{V_{g}\rho_{g}}{V_{ch}\left(Mr_{g}/22.4\right)\left(273/\left(273+T\right)\right)\left(P/101325\right)}$$

where *V_g_* is the gas volume (m^3^) which is calculated from the gas flow rate and the gas exposure time, *ρ_g_* is the relative gas density (g/m^3^), *V_ch_* is the volume of the chamber (m^3^), *Mr_g_* is the molar mass of the gas (g/mol), T is the temperature (°C), and P is the pressure (Pa). Several parts, i.e., two sets of microcantilevers, a temperature-humidity sensor (Sensirion), and cables, were placed inside the chamber. The presence of these parts resulted in a decrease in the effective chamber volume (*V_ch_*) which was predicted to be around 6 × 10^−5^ m^3^. In our case, by inserting the gas into the chamber, the CO concentration gradually rose and then reached saturation after the chamber was filled with the gas. Equation (1) estimates the gas concentration under saturation conditions. In this calculation, for the resonant frequency shift of ∆f~200–400 Hz due to CO with flow rates of 20–100 mL/min, *V_g_* = 5.3 × 10^−5^–5.5 × 10^−4^ m^3^, *ρ_g_* = 1.16 kg/m^3^, *V_ch_* = 6 × 10^−5^ m^3^, *Mr_g_* = 28 g/mol, T = 25 °C, P = 101325 Pa, and the CO concentration for 20–100 mL/min flow rate is approximately 2–13 ppm CO.

## 4. Conclusions

A piezoresistive MC was used to investigate the response of an Al-doped ZnO-nanorod coated MC to CO at room temperature. An increase in the MC resonant frequency, with a sensitivity in the picogram level, was observed, confirming that the MC was capable of monitoring the instantaneous CO concentration in the measuring chamber. The MC could also detect other carbon compound gases, i.e., CO_2_ and CH_4_, though the sensitivities were a half of those observed with CO. These results might lead to the development of novel, highly sensitive CO gas detectors in the future.
